# Human TCR repertoire in cancer

**DOI:** 10.1002/cam4.70164

**Published:** 2024-09-06

**Authors:** Lin Chen, Yuan Hu, Bohao Zheng, Limei Luo, Zhenzhen Su

**Affiliations:** ^1^ Department of Medical Genetics/Prenatal Diagnostic Center West China Second University Hospital, Sichuan University Chengdu China; ^2^ Key Laboratory of Birth Defects and Related Diseases of Women and Children (Sichuan University), Ministry of Education Chengdu China; ^3^ Department of Anesthesia Nursing West China Second University Hospital, Sichuan University/West China School of Nursing, Sichuan University Chengdu China; ^4^ Department of Obstetrics and Gynecology West China Second University Hospital, Sichuan University Chengdu China; ^5^ Wuxi School of Medicine, Jiangnan University Wuxi China; ^6^ Department of Laboratory Medicine West China Hospital of Sichuan University Chengdu China

**Keywords:** cancer, immunotherapy, next‐generation sequencing, T‐cell receptor

## Abstract

**Background:**

T cells, the “superstar” of the immune system, play a crucial role in antitumor immunity. T‐cell receptors (TCR) are crucial molecules that enable T cells to identify antigens and start immunological responses. The body has evolved a unique method for rearrangement, resulting in a vast diversity of TCR repertoires. A healthy TCR repertoire is essential for the particular identification of antigens by T cells.

**Methods:**

In this article, we systematically summarized the TCR creation mechanisms and analysis methodologies, particularly focusing on the application of next‐generation sequencing (NGS) technology. We explore the TCR repertoire in health and cancer, and discuss the implications of TCR repertoire analysis in understanding carcinogenesis, cancer progression, and treatment.

**Results:**

The TCR repertoire analysis has enormous potential for monitoring the emergence and progression of malignancies, as well as assessing therapy response and prognosis. The application of NGS has dramatically accelerated our comprehension of TCR diversity and its role in cancer immunity.

**Conclusions:**

To substantiate the significance of TCR repertoires as biomarkers, more thorough and exhaustive research should be conducted. The TCR repertoire analysis, enabled by advanced sequencing technologies, is poised to become a crucial tool in the future of cancer diagnosis, monitoring, and therapy evaluation.

## 1 INTRODUCTION

Cancer, an immense threat to human health, has an urgent need for more effective methods of diagnosis, monitoring, and treatment.^1^ In recent years, with the successful application of a series of immune checkpoint inhibitors (ICIs) and engineered immune cells in cancer treatment, immunotherapy has become a promising and powerful tool for combating cancer.^2^, ^3^ However, only a subset of people shows effective immune responses to immunotherapy, suggesting that the efficacy of treatment might be influenced by certain factors, especially immune factors.^4^, ^5^ Thus, the identification of cancer immunity and immune characteristics is not only helpful for diagnosis, but also to predict and optimize the therapeutic response of tumor eradication. T cells, which mediate cellular immune response and act as essential components in humoral immune response activation, play a critical role in effective antitumor immune response.^6^ Concerning T cells, it has been found that T cell receptors (TCRs) are crucial molecular markers that determine the specific recognition abilities of T cells.^7^ The collective TCRs of all T cells, known as TCR repertoire, significantly impact almost all health conditions and diseases.^8^ TCR repertoire has been found changing dramatically in cancer progression.^9^, ^10^, ^11^ Therefore, the analysis or evaluation of TCR repertoire in cancers will assist in identifying cancer immunity and immune characteristics, promoting the development of diagnostic and monitoring tools, and effective immunotherapies. Here, we first introduced the TCR repertoire and detection methods and reviewed some of the insights that have been made using next‐generation sequencing (NGS) technology to profile TCR repertoires in a healthy population. We then went over the applications of TCR sequencing in the diagnosis, monitoring, and therapies of cancer, and we also discussed several obstacles and potentials of this field.

## 2 TCR REPERTOIRE

### 2.1 Generation

**FIGURE 1:**
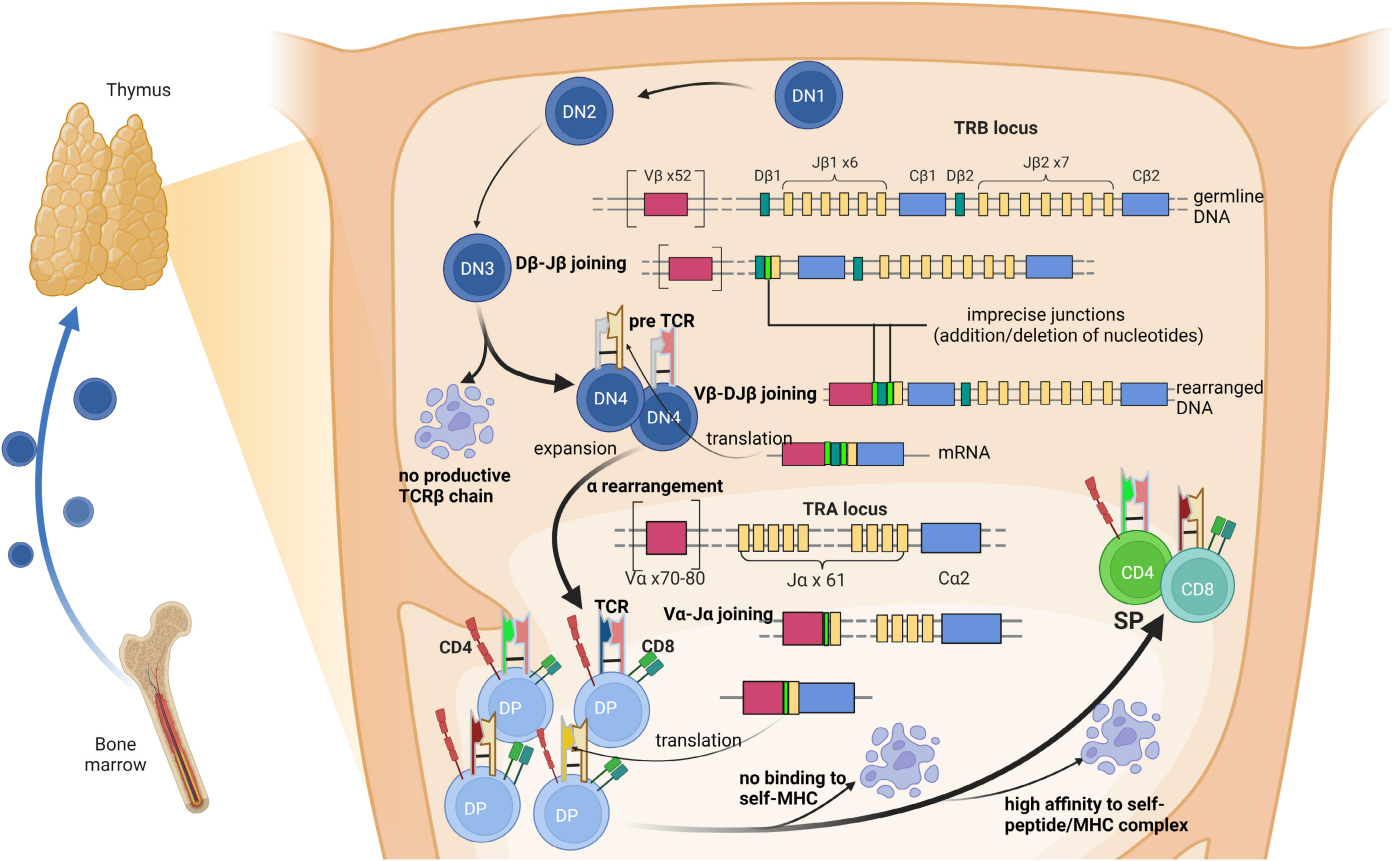
αβT cell development. Lymphoid precursors derived from bone marrow travel to the thymus. These double‐negative (DN) early T cells express neither CD4 nor CD8 and can be subdivided into four stages of differentiation. During lymphocyte development, specificity and diversity result from the V(D)J recombination of distinct variable (V), diversity (D), and joining (J) gene segments. At the DN3 stage, T cell development begins with the joining of D and J gene segments for the TCRβ chain. The V gene segment is joined at the DN4 stage. Each join in the process is preceded by a random insertion or deletion of nucleotides at the junctions. Cells that express a pre TCR consisting of a functional TCRβ chain and an invariant pre TCRα chain proceed to the subsequent stage of development, whereas cells lacking pre TCR undergo apoptosis. The remaining cells undergo ligand‐independent and ligand‐dependent expansion, and TCRα rearrangement including VJ recombination, resulting in expression of TCRs and double positive of CD4 and CD8 (DP). The cells without TCR expression, with TCR that does not recognize self‐major histocompatibility complex (self‐MHC) molecules and with TCR that has excessive affinity for self‐peptide–MHC complex would die (positive selection and negative selection). The remaining cells with TCRαβ and single positive (SP) become naive αβT cells.

On the surface of the T cell membrane, TCR is composed of two disulfide bond‐linked transmembrane proteins: one α chain and one β chain, or one γ chain and one δ chain, which is effectively a heterodimer.^12^, ^13^, ^14^ In the genome, the gene segments of encoding TCR are one of the most complex and broadest loci.^12^, ^15^, ^16^ These loci are found on two chromosomes: β locus is at 7q34, α and δ are at 14q11, and γ is at 7p14.^17^ Unlike other known genes, they inherit in a nonfunctional form and then go a special recombination process, a well‐studied process in immunology field, which is termed V(D)J recombination (see Figure 1). TCR is generated in the thymus. Individual gene segment is selected randomly and separately from the large arrays of variable (V), diversity (D), and joining (J) gene segments in the germline genome and then assembled into the variable region via random rearrangement and imprecise recombination, creating a complete receptor.^12^, ^18^, ^19^, ^20^ The variable region generated by the somatic recombination can encode three complementarity determining regions (CDRs), known as CDR1‐3. CDR1 and CDR2 are encoded by the V gene, while CDR3 is encoded by the junction region VJ (for α or γ) or VDJ (for β or δ), which varies greatly and is in direct contact with antigens.^12^, ^13^, ^21^ After generation of the TCR, thymocytes (immature T cells) expressing TCRs undergo positive selection, which not only ensures that mature T cells can recognize the antigens presented by self‐MHC, but also completes the lineage commitment of CD4/CD8.^22^, ^23^ In this process, only thymocytes whose TCRs can moderately bind to self‐MHC molecules receive survival signals and continue to develop, while those that cannot interact with self‐MHC undergo apoptosis. Meanwhile, interaction with MHC class I molecules leads to differentiation into CD8^+^ T cells, while interaction with MHC class II molecules leads to differentiation into CD4^+^ T cells. Besides, negative selection can eliminates potentially autoreactive T cells that could cause autoimmune diseases and establish immune tolerance.^23^, ^24^ Thymocytes that strongly bind to self‐antigens presented by MHC molecules on thymic dendritic cells and medullary epithelial cells receive apoptotic signals and are eliminated. These processes shape the repertoire that mature T cells can recognize foreign antigens presented by self‐MHC molecules while remaining tolerant to self‐antigens to prevent autoimmunity.^25^, ^26^, ^27^ Given the nearly limitless diversity and rare a priori knowledge of foreign antigens, each of us must develop a repertoire of T cells that could recognize any aberrant protein during life. Fundamentally, this capacity depends on the TCR structural diversity that is the result of V(D)J recombination. In the process of V(D)J recombination, in addition to the diversity of join, each join follows a random insertion or deletion at the junctions, producing an inherent imprecision of join and a more diverse repertoire.^28^, ^29^ Because of the low probability of producing a same V(D)J recombination twice in an individual, the TCR sequence can be used as a unique identifier of T cell clones.^30^ Each clonotype represents a unique TCR variant that can be traced back to a specific T cell clone.^31^ Thus, the study of TCR repertoire is a useful proxy for measuring antigen‐driven clonal expansion of T cells and longitudinal clonal dynamics and heterogeneity of the T cell response. In this review, we refer to TCR repertoire as the collection of TCRs within a given T cell population. Theoretical estimates of TCR types cover a wide range (10^11^ to >10^18^), depending on the assumptions including the length of the nontemplated base sequences inserted at the segment junction and distribution made in diversity calculations.^16^, ^31^, ^32^ The great diversity of TCR represents the biggest challenge in TCR research as there are still technical limitations in obtaining unbiased absolute count data of TCR types.

### 2.2 Detection methods

Some techniques have been used for the analysis of TCR repertoire. Spectratyping, an approach consisting of a CDR3 fragment analysis through capillary electrophoresis, has been applied to screen the TCR repertoire by analyzing peak area and shape of CDR3 profiles.^33^, ^34^ This method can show the degree of TCR skewing (an imbalance in the diversity of the TCR repertoire) and oligoclonality (the presence of a limited number of TCR clones within a population), but not present specific TCR information.^35^ Spectratyping can reveal TCR skewing by showing peaks that represent the overrepresentation of certain TCR clones, leading to an abnormal distribution profile, and demonstrate oligoclonality by showing a restricted set of peaks, each representing a dominant TCR clone, resulting in a narrow and peaked CDR3 distribution profile. Flow cytometry can achieve the information of TCR at protein level by specific anti‐TCR antibodies.^36^ However, the availability of specific antibodies restricts comprehensive analysis of the TCR repertoire. Sanger sequencing can achieve the information of TCR in DNA level up to several 100 cells.^37^ Nonetheless, low‐throughput analysis limits a broader understanding of the TCR repertoire. Over the past decade, the emergence and advancement of NGS technology have provided a robust platform for comprehensive analysis of the TCR repertoire.^38^ NGS is an advanced sequencing technology that allows for the rapid sequencing of large amounts of DNA or RNA. Although the detection process mainly includes sample preparation, PCR amplification, sequencing, and data analysis, DNA sequencing and RNA sequencing differ in terms of the information obtained, their respective applications, complexity, data interpretation, and temporal resolution.^31^, ^39^, ^40^, ^41^, ^42^, ^43^, ^44^ DNA sequencing of TCRs provides information about the potential TCR gene segments present in the genome (germline configuration), which reveals the genetic predisposition for TCR diversity, provides a snapshot of the potential TCR repertoire that an individual or population can generate but does not directly indicate which TCRs are actively being used by T cells. The data interpretation of DNA sequencing of TCRs mainly involves identifying germline gene segments and potential rearrangements, and it is commonly used to study the genetic predisposition and potential diversity of TCRs in an individual or population. RNA sequencing of TCRs focuses on the mRNA transcripts encoding the TCRs, which reflects the TCR sequences that are transcribed and translated into proteins by T cells, providing insights into the actively utilized TCR repertoire and the diversity of T cells responding to specific antigens or conditions. RNA sequencing is usually used to analyze the functional TCR repertoire under specific conditions such as disease states, immune responses, or following treatments like immunotherapy. The data interpretation of RNA sequencing of TCRs is complex due to the variability introduced by post‐transcriptional modifications, alternative splicing, and the need to distinguish between truly expressed TCR sequences and sequencing artifacts. In summary, DNA sequencing of TCRs provides information about the potential diversity encoded in the genome, while RNA sequencing reveals the active repertoire of TCRs being expressed by T cells. Both approaches are valuable for different aspects of understanding immune responses and T cell biology. With the generation of massive sequencing data, new approaches of computational data analysis, as well as data sharing, archiving, and aggregation also developed, making quantitative analysis, diversity delineating, and dynamic evolution tracking of the astronomical TCR repertoire possible.^45^

### 2.3 Characterized metrics

The data set generated by TCR sequencing based on NGS technology usually contains millions of sequences. Several indices have been developed to characterize the TCR repertoire (Table 1). The metrics to characterize TCR repertoire mainly include diversity and clonality. The former reflects the richness of TCR types, while the latter mainly reflects the evenness of TCR distribution.^46^ The changes in TCR repertoire reflect the immune process, and the analysis of these processes will help to understand the antigen‐specific T cell response.^35^ For example, the specific T cell clonal expansion induced by antigen will lead to the change of TCR clonal distribution and the increase of clonality, while the depletion or migration of T cells may lead to the decrease or increase of TCR diversity in the microenvironment where immune response occurs (such as intratumor).^31^ In the published studies, the most commonly used indicators to characterize the diversity of repertoire are based on the Shannon's and Simpson's diversity indices.^47^ The characteristic index realizes the statistical comparisons of TCR repertoire within individuals (such as different time periods) and among individuals, and thus provides a possible hierarchical index for developing a biomarker of the disease monitoring and treatment response, which still needs to be further verified.

**TABLE 1:** Metrics for description and evaluation of TCR repertoire.

index	Calculation formula	Significance
Shannon entropy^48^	$-\sum_{i=1}^{n}p_{i}\log_{e}\left(p_{i}\right)$ *n*, number of clone types; $p_{i}$, the proportion of the i‐th clone relative to the total n clones.	Shannon entropy can evaluate the diversity, reflect the CDR variability and account for both richness and relative abundance. The higher the index, the higher the diversity, the more diverse distribution.
Renyi entropy^49^	$\frac{1}{1-\alpha }\log\left(\sum P_{i}^{\alpha }\right)$ *α*, variable.	Renyi entropy can evaluate the diversity according to the extent of clonal expansion and distribution. It depends on the variable *α*. *α* > 1 means more weight on the abundant species (such as highly expanded TCRs), while *α* < 1 means more weight on the rare ones. It provides more extensive information than a single index because of the considering of differently weighted abundances.
Simpson index^50^	$\sum P_{i}^{2}$ $p_{i}$, the proportion of each productive rearrangement.	Simpson index can evaluate the diversity. High simpson index indicates an uneven distribution of one or a few clones and a less diverse repertoire.
Morisita‐Horn index^51^	$\frac{2p_{1}p_{2}}{p_{1}^{2}+p_{2}^{2}}$ p, the proportion of each clone.	Morisita‐Horn index can evaluate the similarities of repertoires. It is often more suitable to be used in high abundance components, because of the insensitivity to the low abundance components (such as rare TCRs).
Clonality index^52^	$1-\frac{-\sum_{i=1}^{n}p_{i}\log_{e}\left(p_{i}\right)}{\log_{e}\left(n\right)}$ *n*, number of clone types; $p_{i}$, the proportion of the i‐th clone relative to the total n clones.	Clonality index may evaluate clone expansion, indicate the frequency of clone expansion, and measure the difference between two TCR repertoires with varying numbers of clones. It is based on the normalized Shannon entropy, always between 0 and 1. The larger the value, the more clone expansion. The value 1 means monoclonal distribution.
Pielou's evenness index^53^	$\frac{-\sum_{i=1}^{n}p_{i}\log_{e}\left(p_{i}\right)}{\log\left(s\right)}$ *n*, number of clone types; $p_{i}$, the proportion of the i‐th clone relative to the total n clones; s, the number of unique sequences.	Pielou's evenness index permits comparisons between samples with varying total read counts. On a scale from 0 to 1, the higher the score, the more uniform the distribution. The low score indicates clone skewing due to the biased expansion.
High expanded clone (HEC)^54^	$\sum_{i=1}^{n}p_{i}$ *n*, number of clone types; $p_{i}$, the proportion of the i‐th clone relative to the total n clones.	HEC is used to describe the stare of TCR library. It is calculated as the sum of all sequences whose abundance is greater than the threshold. The common threshold is 0.01% or 0.1%, which can be adjusted according to research needs.

## 3 TCR REPERTOIRE IN HEALTH

In humans, blood is the major accessible sample, although it contains only 2%–3% of the total T cells.^55^ There are an estimated 5–10 × 10^9^ T cells in the blood, containing about 1–10 × 10^6^ unique TCR β‐chains, with a similar diversity for TCR α‐chains.^31^, ^56^, ^57^, ^58^, ^59^, ^60^ γδ T cells are detected at frequencies of 1%–5% of T cells in peripheral blood, but the specific diversity of TCR γ or δ chains is not well understood.^61^

The diversity of the TCR repertoire, mainly established during the fetal period and early childhood, is not static throughout life.^62^ Although there is no longitudinal data from cord blood to adulthood, systematic comparisons of TCR repertoires among individuals of different ages indicate that TCR diversity decreases with age. Age‐related contractions in the TCR repertoire are approximately 2–4 fold in healthy elderly subjects compared to young adults.^63^, ^64^, ^65^ It is estimated that 5 × 10^4^ clonotypes of TCR β are lost each year, ranging from 5 × 10^6^ clonotypes in the youngest (6–25 years) to 2.6 × 10^6^ clonotypes in long‐lived donors (71–90 years).^56^ This reduction may be related to age‐related thymic atrophy or involution, which could reduce the output of new naive T cells.^66^, ^67^

Meanwhile, the TCR repertoire is also shaped by an accumulation of “immunization diary” throughout life. In reality, TCR gene recombination in developing thymocytes results in each new naive T cell expressing a unique TCR, and naive T cells predominate in peripheral blood at birth with sizable diversity.^68^, ^69^ When the TCR of a naive T cell binds to a structurally compatible peptide major histocompatibility complex (pMHC) on an antigen‐presenting cell, clones rapidly proliferate and differentiate with the help of costimulatory molecules, generating a population of activated/effector T cells carrying identical TCRs. Normally, after the antigen that triggered the immune response has been cleared, the expanded pool gradually shrinks and persists as a smaller number of memory cells ready for another potential encounter with the antigen. With the gradual accumulation of memory T cells, the TCR repertoire is constantly shaped by immunological challenges and TCR input.^70^ As a result, the overall diversity of the TCR repertoire reduces, which is one of the molecular hallmarks of T cell aging, as also seen in the cancer microenvironment.^71^, ^72^

Another important topic of TCR repertoire studies is public TCRs, which are shared among T cells of multiple individuals. Although the default expectation has been that the TCR repertoire in each individual would be largely unique or private due to high diversity, a large and growing body of research has shown that different individuals often use similar or identical TCRs, at least for one chain of the heterodimer. Previous studies have supported the existence of such public TCRs by showing a similar amount of shared CDR3 clonotypes between monozygotic twins and unrelated babies, and the presence of natural killer T (NKT) and mucosa‐associated invariant T (MAIT) cells with identical TCRα chain sequences in all humans.^73^, ^74^, ^75^ Recently, the overlap has been reported to be 8% for TCRβ chains and 11% for TCRα chains in peripheral blood of three individuals.^76^ The overlap in the thymus is 6.1% and 46.7% for TCRβ and TCRα chains, respectively.^77^ Some studies also focus on the clonal overlap in T cells of different types and locations. For example, a study comparing autoreactive conventional T cells (Tconv) with thymically derived forkhead box protein 3 (FoxP3) + regulatory T cells (tTregs) in human adult and cord blood found virtually no clonotype overlap of TCRβ CDR3.^78^ Another study found an overlap of TCRβ in 5% of naive CD4+ and 3.5% of naive CD8+ T cells, and 2.3% of memory CD4+ and 0.4% of memory CD8+ T cells, respectively.^76^ Comparisons of naive and memory clonotypes in human spleen and lymph nodes found minimal overlap between tissues.^79^ When the total TCR repertoire was evaluated between individuals using standard clinical blood samples, it was observed that less than 0.2% of the repertoires were convergent between individuals.^80^

Currently, several factors are considered to affect clonotype sharing, including convergent recombination (numerous V(D)J recombination events converging to generate the same nucleotide sequence), recombinatorial biases (different gene segments having preferences in usage frequency and pairing), and precursor frequency (different naive T cell precursors being made across a wide range of frequencies).^35^, ^81^, ^82^ In addition, with the discovery of public TCRs enriched in antigen‐experienced populations, antigen‐driven selection is considered to be an important factor in increasing the frequency of these similar clonotypes.^83^, ^84^, ^85^ Although the discovery of antigen‐related public TCRs does not imply that public TCRs are potent or protective species, a growing number of such public TCRs have been found in various immune responses, such as infectious diseases, autoimmunity, and malignancy, suggesting possible virus‐neutralizing activity or other useful functional properties.^86^, ^87^, ^88^ As more and more individuals' repertoires are sequenced in response to specific antigenic exposures, public TCRs will continue to be found. It would be very appealing if the frequency of a particular TCR could predict whether an individual will have a good response to a specific antigen. If this becomes practical in the future, it could be helpful for vaccine development and other planned manipulations of the immune system, such as engineered immune cells for therapeutic intervention in cancer.

## 4 TCR REPERTOIRE IN CANCER

TCR repertoire analysis is invaluable for quantitatively evaluating immune diversity, assisting with early‐stage cancer diagnosis, treatment selection, and prognosis prediction. Profiling TCR repertoires using NGS technologies and associated bioinformatics pipelines has resulted in a powerful tool that can characterize the breadth, strength, and dynamics of the antitumor immune response.

### 4.1 TCR repertoire in solid tumor

In theory, a high diversity of the TCR repertoire increases the possibility of tumor‐specific T cells recognizing corresponding antigens and inhibiting cancer cell growth, thereby limiting immune escape. In reality, significantly decreased TCR repertoire diversity has been observed in peripheral blood of patients with most solid malignancies such as glioblastoma, colorectal cancer, lung cancer, and melanoma.^89^, ^90^ The relationship between decreased TCR diversity and tumorigenesis is unclear. It may be that decreased diversity leads to declined antitumor ability, tumorigenesis consumes specific TCR species, or both. Some studies, such as those finding greater TCR repertoire diversity in the peripheral blood of prostate cancer patients compared to healthy donors, add complexity to this issue.^91^ Based on the characteristics, TCR repertoire analysis could be used to distinguish cancer patients from healthy adults and from patients with other diseases.^92^, ^93^ Further studies have confirmed that the TCR repertoire could serve as a biomarker to monitor cancer immune response.^94^, ^95^, ^96^ In the future, TCR repertoire analysis might become a powerful tool for noninvasive tumor detection and provide insights for tumor detection and monitoring.

Moreover, the TCR repertoire shows a dynamic trend in cancer progression. One study showed that the peripheral TCR diversity decreased while shared clones increased successively in healthy people, patients with cervical intraepithelial neoplasia, early cervical cancer and advanced cervical cancer, indicating that peripheral TCR diversity decreased with cervical cancer progression and that there may be disease‐specific antigen‐mediated immune responses.^97^ Similarly, metastasis‐positive lymph nodes showed a significantly lower TCR diversity and more shared clonotypes compared to metastasis‐negative lymph nodes in colorectal cancer.^98^ In lung cancer, in addition to age‐related decline, peripheral TCR diversity further reduced in patients with larger tumors or more metastatic sites, and the relapsed group exhibited lower diversity than the untreated group.^90^ Dynamic TCR repertoire analysis may serve as a useful indicator of cancer development and guide immunotherapy.

The TCR repertoire is also associated with cancer prognosis. Depending on the cancer type, both high and low diversity in tumor tissue or blood TCR repertoire can be associated with better prognosis. For instance, in melanoma, patients with late relapse and long progression‐free survival (PFS) showed significantly higher TCR repertoire diversity in blood than those with rapid progression and poor prognosis.^99^ In gastric cancer, low diversity of TCR repertoire within the tumor‐adjacent mucosal tissue is associated with a poor clinical prognosis in patients, and the mucosal TCR repertoire diversity index is an independent predictor for survival of patients.^100^ In lung cancer, the higher TCR repertoire diversity in tumor tissues is associated with worse cancer outcomes, while patients with higher TCR diversity in peripheral blood shows a longer PFS.^90^, ^101^ In cervical cancer, less clonotypes in the TCR repertoire of sentinel lymph node was associated with the poor prognosis.^97^ In mycosis fungoides, the high TCR clonality in tumors is related to the decrease of PFS and overall survival.^102^ The relationship between TCR diversity and cancer is summarized in Table S1. These findings highlight the need for more precise studies on specific tumor types.

Analysis of public TCR clones amplified in primary and metastatic tumors can enhance understanding of antigen‐specific TCRs and predict prognosis.^103^ Notably, considering the ease and noninvasiveness of peripheral blood sampling, most studies are based on peripheral blood samples, which may not accurately reflect the tumor microenvironment. Some studies have extended the analyses beyond peripheral blood, showing significant differences between intratumoral and peripheral blood TCR repertoires.^101^, ^104^, ^105^, ^106^, ^107^ More evidence suggests TCR repertoire heterogeneity, differing in various cancers and within different regions of the same tumor.^103^, ^108^, ^109^ On the one hand, it is due to the differences in immune microenvironment in different cancer types. A group of expanded T cell clones which may spatially confine to the tumor microenvironment mainly reacting to tumor antigens. On the other hand, it may be related to tumor neoantigens. This view is supported by number of studies that reported TCR heterogeneity landscape in both primary and metastatic tumors.^109^, ^110^, ^111^, ^112^ TCRs present in tumors or other regions may mainly represent the response to tumor antigens found in cancer cells. Because neoantigens can gradually appear in the process of tumor evolution, the number of neoantigens in different regions of the tumor may vary greatly, which contributes to the spatial heterogeneity in TCR repertoire. In turn, TCR heterogeneity can reflect specific selective amplification of TCR in tumors and is related to the number of somatic mutations leading to tumor heterogeneity.^113^, ^114^ In lung adenocarcinoma, higher TCR intratumor heterogeneity is associated with higher predicted neoantigen heterogeneity, higher risk of postsurgical relapse, and shorter disease‐free survival, suggesting potential clinical significance of TCR repertoire heterogeneity.^112^

### 4.2 TCR repertoire in nonsolid tumor

Patients with hematological (but non‐T cell) cancers, such as acute myeloid leukemia (AML), showed significantly higher clonality and lower diversity of blood TCR repertoire, even after excluding the age effects.^89^, ^115^ In diffuse large B‐cell lymphoma (DLBCL), the TCR diversity within DLBCL nodes is abnormally narrow relative to nonpathological node tissues.^116^ This change is expected due to the infiltration of primary and/or secondary lymphoid organs in these patients, which potentially hinder the emigration of hematopoietic precursors to the thymus and/or peripheral T cell expansion. In T lymphoid malignancy, TCR shows highly clonal expansion and significantly decreased diversity.^117^ As TCR repertoire could reflect the immunological status and abnormality, TCR repertoire analysis based on NGS can be used to detect malignant clones, identify clonal amplification and track minimal residual disease (MRD) in some blood disorders, demonstrating better sensitivity and accuracy than conventional methods.^39^, ^93^, ^118^, ^119^ Moreover, more obvious clonal amplification is observed in patients with AML recurrence than in the first diagnosis.^120^ Similarly, high TCR clonality in DLBCL associates with poor outcome in conventional frontline therapy.^116^ These suggest that TCR repertoire analysis may be related to the progression and prognosis, which needs further exploration.

## 5 TCR REPERTOIRE DURING CANCER TREATMENT

Cancer poses a significant threat to human health, and its treatment has always been a major focus. Traditional anticancer therapies such as surgical intervention, chemotherapy, and radiotherapy mainly focus on removing or killing tumor cells directly. With the success of immunotherapy, represented by immune checkpoint inhibitors (ICIs) in improving cancer patient survival, recent researches on cancer treatment pay a large of attention to the anticancer effect of the immune system.^121^ As crucial factors of the anticancer immune response, T cells affect and participate in the process of immunotherapy and traditional treatments.^122^, ^123^ In addition, detecting tumor‐specific or neoantigen‐specific TCRs is a key step that directly affects treatment efficacy.^124^ Thus, in‐depth analysis of the TCR repertoire has a potential to provide necessary insights for understanding individual tumor and immunity and guide treatment.

### 5.1 TCR repertoire in traditional treatments

For most solid tumors, timely surgical intervention is the first tier treatment. In patients with colorectal cancer, peripheral TCR expansion clone were higher than those in healthy controls, but decreased after surgery.^54^ In resectable non‐small cell lung cancer, an association was observed between higher intratumoral TCR clonality and reduced percent residual tumor at the time of surgery.^125^ The change of TCR repertoire after surgery, on the one hand, is due to the removal of tumor; on the other hand, it may also be related to the effect of surgical stress on immune state.^126^ Although the reasons for these changes are complex, TCR repertoire can characterize the overall immune state to some extent and has potential for predicting surgery prognosis.

Radiotherapy and chemotherapy are not only the main treatment of nonsolid tumors, but also often combined with surgery to treat solid tumors. Experimental models indicate radiation therapy increases TCR diversity, which contributes to response.^122^ TCR repertoire analysis in two cohorts (glioblastoma and chronic lymphocytic leukemia) with paired pre‐ and post‐treatment blood samples showed that there was no drastic change in diversity or clonality after chemotherapy, but there was almost no clonal overlap, that is, TCR remodeling after chemotherapy changes the clone type.^89^ Surprisingly, even in elderly cancer patients, age‐specific metrics of repertoire can be completely restored after T cell lymphotoxic chemotherapy. It suggests that it may be unnecessary to worry about the drastic and uncompensated loss of diversity in the elderly population caused by chemotherapy, which opens up an interesting new perspective on immunosenescence and immune reconstruction in cancer patients, and may affect the way we think about immunologic and cytotoxic cancer treatment. In addition, a severe restricted diversity or highly clonality of peripheral blood TCR repertoire at baseline was reported to associate with poor outcome treated with chemotherapy.^116^, ^127^, ^128^ Thus, significantly reduced age‐adjusted TCR diversity in cancer patients may indicate TCR repertoire defects, which could be both a consequence and a potential basis for carcinogenesis.

### 5.2 TCR repertoire in immunotherapy

Immunotherapeutic strategies activate the immune system and boost tumor‐specific immune responses. The most effective cancer immunotherapies currently include ICIs, tumor‐specific antigen‐based cancer vaccines, adoptive cell transfer (ACT), and antibody‐dependent cellular cytotoxicity (ADCC).^129^, ^130^, ^131^, ^132^, ^133^, ^134^ However, not all patients show clinical improvement from immunotherapy, and adverse events can occur.^135^, ^136^ At present, there are few biomarkers that can be used to predict clinical benefit and monitor post‐treatment immune response. Although the relationship between TCR repertoire and ACT or ADCC therapy response is unclear, studies have shown that TCR repertoire analysis has great potential in ICIs and cancer vaccine immunotherapies.

#### 5.2.1 ICIs

As one of the representatives of immunotherapy, ICIs have become a powerful clinical strategy for combating cancer. The blockade of two vital molecules for T cell related immune response, cytotoxic T lymphocyte‐associated antigen 4 (CTLA‐4) and programmed cell death protein 1 (PD‐1), have been approved for clinical application. Despite a subset of cancer patients has obtained unprecedented clinical benefits from this treatment, the majority of patients across different cancers does not benefit from this costly therapy. It is not clear how ICIs induce persistent tumor response or autoimmune toxicity. The clinical need for biomarkers that predict the likelihood of patients benefiting from these drugs before or early treatment has also not been met. The TCR repertoire analysis in the process of immune checkpoint blockade may provide insights into the mechanisms, and further predict clinical benefits and outcomes.

##### Anti‐CTLA‐4

Universal increased peripheral blood TCR diversity after anti‐CTLA‐4 therapy is reported.^53^, ^137^, ^138^, ^139^ This may be attributed to the fact that CTLA‐4 is a checkpoint involved in the early initiation stage of immune response. However, the relationship between TCR repertoire and clinical response is controversial. Some studies showed that the peripheral diversification is irrespective of clinical responses for anti‐CTLA‐4 treatment,^53^, ^140^ while other studies suggested TCR repertoire features of patients could potentially be used as a predictive biomarker for clinical response.^137^, ^139^, ^141^, ^142^, ^143^, ^144^ The differences of CTLA‐4 blocking antibodies (such as tremelimumab and ipilimumab), sample type and detection technology may be the reasons for the different results about the relationship between TCR repertoire and clinical responses. Significantly, the TCR repertoire is highly different before and after anti‐CTLA‐4 treatment, and the overlap is low.^53^, ^139^ This suggests that anti‐CTLA‐4 therapy can promote a rapid influx of new T cell clones and broaden the circulating TCR repertoire. However, this broadening is nonspecific because the increased diversity of peripheral TCR has an association with anti‐CTLA‐4 toxicity, which in part reflect checkpoint inhibition may also result in mobilization of auto‐reactive T cells.^53^, ^138^

##### Anti‐PD‐1

Increased peripheral TCR diversity was also found in tumor patients after anti‐PD‐1 treatment.^141^ However, more studies have shown that anti‐PD‐1 treatment is mainly related to TCR clonal expansion rather than diversification, suggesting that anti‐PD‐1 may be more conducive to the maintenance and expansion of existing antitumor T cells.^145^, ^146^, ^147^, ^148^, ^149^ Considering the inverse association between TCR clonality and percent residual tumor at the time of surgery, it provides a justification for the use of anti‐PD‐1 before surgery.^125^ Unlike anti‐CTLA‐4, the clonality of TCR repertoire shows a close relationship with the clinical response of anti‐PD‐1 treatment. A high clonality in pretreatment relates to a longer progression‐free survival and better response to anti‐PD‐1 therapy.^145^, ^146^, ^150^ Meanwhile, following PD‐1 blockade therapy, the clonality increase has been found to associate with clinical response in different cancers, such as melanoma, non‐small cell lung cancer, pancreatic cancer, and merkel cell carcinoma.^139^, ^147^, ^151^, ^152^ Thus, it may be feasible to assess the response of checkpoint blockade before or early treatment by monitoring the TCR repertoire.

#### 5.2.2 Cancer vaccines

Therapeutic cancer vaccines usually include selected tumor antigens, immune activating adjuvants, and even immune cells such as dendritic cells (DCs).^153^ Their goal is to induce tumor regression, eradicate MRD, establish lasting antitumor memory, and avoid nonspecific or adverse reactions. However, tumor‐induced immunosuppression and immune resistance pose a major challenge to achieve this goal. TCR repertoire analysis could provide a better understanding of the breadth, intensity, and dynamics of antitumor immune response, which will help to improve vaccine design.

Sipuleucel‐T, an FDA approved DC‐focused vaccine, has been reported to promote the recruitment of T cells into prostate tissue and increase the diversity of TCR in the tumor tissue, so as to enhance antitumor immunity.^91^ Similarly, other vaccines change the clonality or/and diversity of TCR repertoire, as well as the neoantigen‐specific immunity in cancers, including glioma, melanoma, lung cancer, and mesothelioma.^154^, ^155^, ^156^, ^157^ Moreover, the clinical response to cancer vaccine is closely related to the characteristics of TCR repertoire before and after treatment.^156^, ^157^ Patients with high clonality of TCR repertoire before immunotherapy and increased diversity after vaccine treatment show a better clinical response, which is similar with anti‐CTLA4 or anti‐PD1 treatment in other cancers.^137^, ^147^ It seems that the presence of circulating expanded TCR clones is generally beneficial for the clinical response to immunotherapy. However, it is not clear whether these clones reflect a pre‐existing tumor‐specific TCR. It is worth noting that although the change trend of TCR repertoire after using anti‐CTLA‐4 or anti‐PD‐1 is similar to that after vaccine treatment, clinical researches on mesothelioma using anti‐CTLA‐4 or anti‐PD‐1 were unsuccessful or little effective,^158^, ^159^, ^160^ suggesting that different immunotherapy approachws may target a certain type of T cell subsets, and the change of single subset will affect the TCR repertoire analysis. Therefore, further identification of TCR clonetypes associated with immunotherapy will contribute to predict treatment‐related clinical outcomes.

The success of cancer immunotherapy depends on the inducing and producing effective tumor specific immune response. The combination of different immunotherapy methods (such as cancer vaccine and ICIs) may synergistically enhance antitumor response and optimize the potential of immunotherapy.^161^ In order to further improve the effect of immunotherapy, a deeper understanding of host‐tumor interaction and tumor immune escape strategy is required. The existing researches show that the diversity and clonality of TCR repertoire are closely related to the clinical responses to immunotherapy, providing insights into the mechanisms. As immunotherapy act on the immune components rather than tumor cells directly, it is likely that the same features of the TCR repertoire could be generalized to predict response to immunotherapy for most patients eligible for this treatment. A broader research scope, larger sample sizes, and prospective validation are expected to determine whether TCR repertoire analysis can be used as a biomarker for stratification and monitoring of immunotherapy, so as to prognosticate the clinical benefits.

## 6 FUTURE DIRECTIONS

Overall, TCR repertoire appears to be a biomarker of antitumor adaptive immunity and might also be predictive markers of responses to cancer immunotherapy. Although TCR repertoire analysis of tumor microenvironment may better reflect antitumor immunity, blood samples will be the preferred samples for analysis because they are easy to obtain and noninvasive. Therefore, it is necessary to explore the TCR repertoire consistencies and differences between peripheral blood, tumoral, and adjacent normal tissues. The dynamic characteristics of TCR repertoire in different cancers also deserve further discussion. In addition to monitoring, we expect that TCR repertoire analysis can also determine whether clonally expanded T cells actually recognize tumor specific antigens in individual patients, and then be used to create tumor reactive T cells.

Due to CDR3 of TCRβ Chain represents the most diversity of TCR repertoire, most studies have focused on it. Although sequence of TCRβ can be used as a unique identifier for a specific T cell, it cannot be used alone to determine the antigen specificity or tumor reactivity of a specific T cell. It may be helpful to predict the peptide specificity of TCR by adjusting the method to obtain the information of both chains of TCR. At the same time, the γδTCR deserves more research.

Moreover, the analysis of TCR repertoire should be more combined with the phenotypic status of T cells. The current studies mainly focuses on the analysis of molecular characteristics, without combining the staining and sorting information of T cell markers to obtain more detailed information about T cell clonotypes. The changes of different T cell subtypes may lead to the same TCR repertoire changes, but their actual immune effects are different. For example, clonal expansion of regulatory T cells is certainly more detrimental for patients than that of cytotoxic T cells. Therefore, the combination of TCR sequencing and T cell phenotypic characteristics will be more conducive to explore the antitumor immunity.

It should be further noted the opening up of novel approaches to study TCR repertoire. The index of diversity or clonality mainly reflects the distribution of repertoire (such as richness and evenness). The analysis methods or indicators focusing on TCR functions are worthy of efforts. For example, based on the assumption that TCRs that reacted to the same antigen have similar CDR3 amino acid sequences, recent studies have developed cluster analysis of highly similar sequences, providing biomarkers for tumor monitoring and treatment response that are more powerful than other indexes.^162^, ^163^, ^164^ Some new computing methods, especially machine learning algorithms, also provide new possibilities for predicting TCR antigen specificity.^165^ The new methods that will properly solve the current challenges in TCR repertoire analysis of cancers are highly anticipated.

## 7 CONCLUSION

TCR repertoire analysis is increasingly becoming an indispensable weapon in cancer immune research. With the further decline of sequencing cost, the more standardized protocols, and the more reliable and user‐friendly computational tools, TCR repertoire analysis will be more and more used in clinical context, including monitoring the occurrence and progression of diseases, evaluating the response to treatment, patient stratification, prognosis evaluation and so on. In addition, with the development and application of computational methods and artificial intelligence, the most powerful impact of TCR repertoire analysis may be to annotate the antigen‐specific repertoire in antitumor immune response, identifying tumor neoantigens and T cells that respond to tumor antigens. By then, cancer immunotherapy such as adoptive cellular therapy and cancer vaccine will enter a new era.

## AUTHOR CONTRIBUTIONS

**Lin Chen:** Formal analysis (lead); writing – original draft (lead). **Yuan Hu:** Writing – original draft (supporting). **Bohao Zheng:** Formal analysis (supporting); writing – original draft (supporting). **Limei Luo:** Formal analysis (supporting). **Zhenzhen Su:** Conceptualization (lead).

## CONFLICT OF INTEREST STATEMENT

The authors declare that they have no conflict of interest.

## Supporting information


Table S1.

## Data Availability

Not applicable.
