# Supplementary material for: Human TCR repertoire in cancer
Source: Cancer Med. 2024 Sep 6;13(17):e70164. doi: 10.1002/cam4.70164 (PMC11378360; doi:10.1002/cam4.70164)
Supplement: Supplementary file 1 — Table S1. [file CAM4-13-e70164-s001.docx]

| **Tumor Type** | **Sample** | **TCR Diversity** | **Relationship** | **Reference** |
| --- | --- | --- | --- | --- |
| **Lung Cancer** | PBMC | Increased | Higher TCR diversity in peripheral blood correlates with better prognosis and better outcomes after immunotherapy. | 1-4 |
| **Breast Cancer** | PBMC | Decreased | Lower TCR diversity is associated with poorer prognosis and resistance to therapies. | 5,6 |
| **Cervical Cancer** | PBMC | Increased | Increased TCR diversity is linked to better immune response and improved prognosis. | 7 |
| **Liver Cancer (HCC)** | Tumor tissue | Decreased | Reduced TCR diversity is correlated with advanced disease and poor prognosis. | 8-11 |
| **Prostate Cancer** | PBMC | Decreased | Lower TCR diversity is often associated with disease progression and poor outcomes. | 12,13 |
| **Gastric Cancer** | PBMC/ tumor tissue and adjacent mucosa | Increased | Higher TCR diversity of mucosal T is linked to better response to immune checkpoint inhibitors and improved prognosis. | 14 |
| **Colorectal Cancer** | Tumor tissue and adjacent healthy mucosa | Increased | Increased TCR diversity is associated with better response to immunotherapy and overall improved prognosis. | 15,16 |
| **Melanoma** | PBMC/ adjacent tissue | Increased | Higher TCR diversity is associated with better outcomes and long-lasting clinical responses to PD-1 targeted therapy. | 12,17-23 |
| **Metastatic Melanoma** | PBMC/ adjacent tissue | Increased | A polyclonal TCR repertoire directs against a small number of cancer-specific targets enhances the success of immunotherapy. | 23 |
| **Glioma** | PBMC/ tumor tissue / nonneoplastic brain tissue | Decrease | Diversity and divergence of glioma-infiltrating TCR repertoire links to poorer outcomes. | 24 |
| **Nasopharyngeal carcinoma** | PBMC/ tumor tissue / adjacent normal tissue | Decrease | Higher TCR diversity in peripheral blood is associated with a worse prognosis. Lower TCR diversity of in tumors is associated with a poor prognosis. | 25 |
| **Pancreatic ductal adenocarcinoma** | PBMC | Increased | Higher TCR diversity correlates with better prognosis after immunotherapy. | 26 |
| **Urothelial Carcinoma** | PBMC | Increased | Higher TCR diversity correlates with better prognosis after immunotherapy. | 27 |

**Table S1. The association between TCR diversity and cancer**

PBMC, peripheral blood mononuclear cells; TCR, T cell receptor; HCC, hepatocellular carcinoma.

**References**

1. Han J, Duan J, Bai H, et al. TCR Repertoire Diversity of Peripheral PD-1+CD8+ T Cells Predicts Clinical Outcomes after Immunotherapy in Patients with Non-Small Cell Lung Cancer. *Cancer Immunol Res*. 2020; 8(1):146-154.

2. Liu YY, Yang QF, Yang JS, et al. Characteristics and prognostic significance of profiling the peripheral blood T-cell receptor repertoire in patients with advanced lung cancer. *Int J Cancer*. 2019, 145(5): 1423-1431.

3. Naidus E, Bouquet J, Oh DY, et al. Early changes in the circulating T cells are associated with clinical outcomes after PD-L1 blockade by durvalumab in advanced NSCLC patients. *Cancer Immunol Immunother*. 2021; 70(7): 2095-2102.

4. Joshi K, De Massy MR, Ismail M, et al. Spatial heterogeneity of the T cell receptor repertoire reflects the mutational landscape in lung cancer. *Nat Med*. 2019, 25(10): 1549-1559.

5. Manuel M, Tredan O, Bachelot T, et al. Lymphopenia combined with low TCR diversity (divpenia) predicts poor overall survival in metastatic breast cancer patients. *Oncoimmunology*. 2012; 1(4): 432-440.

6. Page DB, Yuan J, Redmond D, et al. Deep Sequencing of T-cell Receptor DNA as a Biomarker of Clonally Expanded TILs in Breast Cancer after Immunotherapy. *Cancer Immunol Res*. 2016; 4(10): 835-844.

7. Cui JH, Lin KR, Yuan SH, et al. TCR Repertoire as a Novel Indicator for Immune Monitoring and Prognosis Assessment of Patients With Cervical Cancer. *Front Immunol.* 2018; 9: 2729.

8. Chen Y, Xu Y, Zhao M, et al. High-throughput T cell receptor sequencing reveals distinct repertoires between tumor and adjacent non-tumor tissues in HBV-associated HCC. *Oncoimmunology*. 2016; 5(10): e1219010.

9. Han Y, Liu X, Wang Y, et al. Identification of characteristic TRB V usage in HBV-associated HCC by using differential expression profiling analysis. *Oncoimmunology*. 2015; 4(8): e1021537.

10. Han Y, Li H, Guan Y, Huang J. Immune repertoire: A potential biomarker and therapeutic for hepatocellular carcinoma. *Cancer Lett*. 2016; 379(2): 206-212.

11. Lin KR, Deng FW, Jin YB, et al. T cell receptor repertoire profiling predicts the prognosis of HBV-associated hepatocellular carcinoma. *Cancer Med*. 2018; 7(8): 3755-3762.

12. Cha E, Klinger M, Hou Y, et al. Improved survival with T cell clonotype stability after anti-CTLA-4 treatment in cancer patients. *Sci Transl Med*. 2014; 6(238): 238ra70.

13. Sheikh N, Cham J, Zhang L, et al. Clonotypic Diversification of Intratumoral T Cells Following Sipuleucel-T Treatment in Prostate Cancer Subjects. *Cancer Res.* 2016; 76(13): 3711-3718.

14. Jia Q, Zhou J, Chen G, et al. Diversity index of mucosal resident T lymphocyte repertoire predicts clinical prognosis in gastric cancer. *Oncoimmunology*. 2015; 4(4): e1001230.

15. Sherwood AM, Emerson RO, Scherer D, et al. Tumor-infiltrating lymphocytes in colorectal tumors display a diversity of T cell receptor sequences that differ from the T cells in adjacent mucosal tissue. *Cancer Immunol Immunother*. 2013; 62(9): 1453-1461.

16. Matsuda T, Miyauchi E, Hsu YW, et al. TCR sequencing analysis of cancer tissues and tumor draining lymph nodes in colorectal cancer patients. *Oncoimmunology*. 2019; 8(6): e1588085.

17. Charles J, Mouret S, Challende I, et al. T-cell receptor diversity as a prognostic biomarker in melanoma patients. *Pigment Cell Melanoma Res.* 2020; 33(4): 612-624.

18. Hogan SA, Courtier A, Cheng PF, et al. Peripheral Blood TCR Repertoire Profiling May Facilitate Patient Stratification for Immunotherapy against Melanoma. *Cancer Immunol Res*. 2019; 7(1): 77-85.

19. Postow MA, Manuel M, Wong P, et al. Peripheral T cell receptor diversity is associated with clinical outcomes following ipilimumab treatment in metastatic melanoma. *J Immunother Cancer*. 2015; 3: 23.

20. Khunger A, Rytlewski JA, Fields P, Yusko EC, Tarhini AA. The impact of CTLA-4 blockade and interferon-α on clonality of T-cell repertoire in the tumor microenvironment and peripheral blood of metastatic melanoma patients. *Oncoimmunology*. 2019; 8(11): e1652538.

21. Tumeh PC, Harview CL, Yearley JH, et al. PD-1 blockade induces responses by inhibiting adaptive immune resistance. *Nature*. 2014; 515(7528): 568-571.

22. Yusko E, Vignali M, Wilson RK, et al. Association of Tumor Microenvironment T-cell Repertoire and Mutational Load with Clinical Outcome after Sequential Checkpoint Blockade in Melanoma. *Cancer Immunol Res.* 2019; 7(3): 458-465.

23. TCR Diversity Underpins Immunotherapy Success. *Cancer Discov*. 2023; 13(5): 1031-1032.

24. Sims JS, Grinshpun B, Feng Y, et al. Diversity and divergence of the glioma-infiltrating T-cell receptor repertoire. *Proc Natl Acad Sci U S A*. 2016; 113(25): E3529-E3537.

25. Jin YB, Luo W, Zhang GY, et al. TCR repertoire profiling of tumors, adjacent normal tissues, and peripheral blood predicts survival in nasopharyngeal carcinoma. *Cancer Immunol Immunother*. 2018; 67(11): 1719-1730.

26. Hopkins AC, Yarchoan M, Durham JN, et al. T cell receptor repertoire features associated with survival in immunotherapy-treated pancreatic ductal adenocarcinoma. *JCI Insight*. 2018; 3(13): e122092.

27. Snyder A, Nathanson T, Funt SA, et al. Contribution of systemic and somatic factors to clinical response and resistance to PD-L1 blockade in urothelial cancer: An exploratory multi-omic analysis. *PLoS Med*. 2017; 14(5): e1002309.
